# The main indicators for Iranian hospital ethical accreditation

**Published:** 2015-07

**Authors:** SEYED ALI ENJOO, MITRA AMINI, SEYED ZIAADIN TABEI, ALI MAHBUDI, ZAHRA KAVOSI, MAHBOOBEH SABER

**Affiliations:** 1 Medical Ethics Department, Medical School, Shiraz University of Medical Sciences, Shiraz, Iran;; 2 Quality Improvement in Clinical Education Research Center, Shiraz University of Medical Sciences, Shiraz, Iran;; 3 Social Determinants of Health Research Center, Shiraz University of Medical Sciences, Shiraz, Iran

**Keywords:** Hospital, Ethics, Accreditation, Evaluation

## Abstract

**Introduction:**

The application of organizational ethics in hospitals is one of the novel ways to improve medical ethics. Nowadays achieving efficient and sufficient ethical hospital indicators seems to be inevitable. In this connection, the present study aims to determine the best indicators in hospital accreditation.

**Methods:**

69 indicators in 11 fields to evaluate hospital ethics were achieved through a five-step qualitative and quantitative study including literature review, expert focus group, Likert scale survey, 3 rounded Delphi, and content validity measurement. Expert focus group meeting was conducted, employing Nominal Group Technique (NGT). After running NGT, a three rounded Delphi and parallel to Delphi and a Likert scale survey were performed to obtain objective indicators for each domain. The experts were all healthcare professionals who were also medical ethics researchers, teachers, or PhD students. Content validity measurements were computed, using the viewpoints of two different expert groups, some ethicists, and some health care professionals (n=46).

**Results:**

After conducting NGT, Delphi, Likert survey, 11 main domains were listed including:  Informed consent, Medical confidentiality, Physician-patient economic relations, Ethics consultation policy in the hospital, Ethical charter of hospital, Breaking bad medical news protocol, Respect for the patients’ rights, Clinical ethics committee, Spiritual and palliative care unit programs in the hospitals, Healthcare professionals’ communication skills, and Equitable access to the healthcare. Also 71 objective indicators for these 11 domains were listed in 11 tables with 5 to 8 indicators per table. Content Validity Ratio (CVR) measurements were done and 69 indicators were highlighted.

**Conclusion:**

The domains listed in this study seem to be the most important ones for evaluating hospital ethics programs and services. Healthcare organizations’ accreditation and ranking are crucial for the improvement of healthcare services. Ethics programs would also motivate hospitals to improve their services and move towards patients’ satisfaction. In this regard, more involvement of bioethicists can help healthcare organizations to develop ethics programs and ensure ethics-based practice in hospitals.

## Introduction

Some bioethics experts argue that the scopes of ethics in healthcare professions are divided into theoretical (philosophical) and applied branches. Regarding the former, the focus is mainly on theories (1), norms (2), principles (2, 3), virtues (2), etc. However, the major domains in the latter such as confidentiality, abortion, and euthanasia are mostly concerned with individual healthcare behaviour (4). Organizational ethics is the new approach to medical ethics focusing on healthcare organizations behaviour. Its novelty is so much that some experts have called it the ‘next step’ in medical ethics (5) or “the next edge of bioethics to push on”. This next step of bioethics requires reorientation of clinical ethics from issues concerning the individual patient to the wider sociological background. As Potter maintains bioethics applies clinical ethics in the first stage of evolution and it is the time to get fully involved in organizational ethics (6). Suhonen et al. argue that organizational ethics ought to be maintained alongside the growing awareness of patients’ rights and a strengthening of regulatory systems, legislation and health policy in health care in recent years (7). One of the most important organizations in health care is hospital. As a result, an important step in the development of medical ethics is the improvement of hospital ethics. For this purpose, there is a need for ethical standard indicators. There are different ways to improve ethics in hospitals. One way is to equip professionals such as physicians, nurses (8), managers (9) and so on with the necessary and required ethical standards. On the other hand, some of the medical ethicists are trying to improve hospital ethics by ethical committees (10) and ethical consultation or conducting code of ethics for the hospitals (e.g. the longstanding guidelines on ethical conduct and relationships for health care institutions of the American Hospital Association (AHA), (The first version was issued in 1973)) (11). The development of hospitals from small facilities into very large organizations has motivated "smaller organizations to seek the shelter of large organizations" (6). Spencer et al. hold that healthcare organizational behaviour reflects the organizational intrinsic and extrinsic values. By programming and evaluating those organizational behaviours through useful guidelines, we can develop positive ethical climate and culture in the organization (12). Some organizations such as JCAHO are now working on the matter (13). Nowadays hospital ethical accreditation is a novel hot topic (14), but the domains seem not to be limited only to the mentioned ethical accreditation domains. In spite of some differences among the main ethical domains in different cultures because of dependency of ethics on culture (15), there are a lot of common ethical values so that we could develop some global standards for hospital ethical accreditation. In teaching hospitals,  there is an interaction among the students and the environment within which the students develop professionalism. Medical personnel should have a unique combination of different abilities. Harden et al. designed a three-circle model for doctors' responsibilities. The internal circle included performance of tasks (Technical intelligences), the middle circle approaches to tasks (Intellectual and creative intelligences), and the outer circle the growing of the individual (Professionalism: Personal intelligences) (16). JCAHO developed the first standards manual containing 18 pages in 1926, and more than 3,200 hospitals attained accreditation based on this manual until 1950. The standards are concerned with Ambulatory Health Care, Behavioural Health Care, Critical Access Hospitals, Home Care (+Pharmacy), Hospital, Laboratory Services, Nursing Care Centre, and International Accreditation (13). Min-Hua et al. raised a question on integrated ethical and medical accreditation or distinct ethical and medical accreditations (14). Thomas et al. have shown a paradigm shift in managing health care system in recent years. They maintain that the paradigm changing has occurred not only in the medical care services, but also in the system management mission. They also argue that health care system goals vary from individual and short term objectives to the systemic and organizational ones. Changes lead to integrated health care delivery systems/networks (IDSs), causing more clinical and administrative efficiency, fewer unneeded services, higher profits, increased market power, negotiation power, environmental acceptance, relationships with customers and improved quality of care (17). In the past decades, accreditation of medical residency programs aiming at improving the quality of medical care was performed (18). JCAHO was the pioneer in responding to the necessity of setting standards related to the patients’ rights and organizational bioethics. Codman proposed the “end result system of hospital standardization” in 1910. That system was the minimum standards hospitals required to observe patients’ rights. The aim was to achieve the optimal achievable levels of quality, and this was achieved in 1970 (13). Potter maintains that this is only the first sketch of the comprehensive picture of the organizational ethical standards to be drawn (6). The Accreditation Council for Graduate Medical Education (ACGME), proposing improvement of patient safety, intern and resident’s life and education, established some national regulatory programs for this purpose (19). Commons and Baldwin developed an ethical policy guideline for evaluating general nurses. They focused on general hospital nurses’ ethical behaviour (20). Although the capacity building had started long ago (21, 22), Iran began improving ethics in the health care in 2008 by establishing Ph.D of medical ethics in 3 universities. On the other hand, clinical quality improvement protocols such as clinical governance and accreditation are running in the country with some of the ethical criteria (23, 24). Currently, parallel efforts to improve quality of patient care are made in Iran (25). Major ethical challenges vary from one society to another, so if we want to improve our hospital organizational ethics, it is necessary to have objective indicators. Regarding all this, developing ethical standards for improving hospital ethics seems to be crucial. In Iran there is a good communication between health system hospitals and medical schools (26, 27).

Every organization requires a clear statement of quality management. Hospitals are the organizations dealing with human beings, so ethical standards in such organizations seem to be very important. Unfortunately, many of these organizations don't usually realize the importance of setting ethical standards in their routine, quality assurance programs. Today the medical ethics community is trying to develop more effective approaches for quality improvement in medical care ethics (28).

Without setting standards and a common focus, the achievement of significant ethical goals, which is one of proposes of the Ministry of Health and Medical Education (29), is nearly impossible for Iranian hospitals. In this connection, the main objective of this research was to define and list the main domains and indicators to evaluate ethics in Iranian hospitals. In fact, the objectives were: 1) to identify the current important domains in hospital medical ethics as described by a group of experts in NGT, 2) to prioritize these domains based on the NGT, Delphi and Likert survey, 3) to list the indicators for each domain based on a three rounded Delphi and 4) to do content validity testing.

## Methods

The study, which is both qualitative and quantitative, was conducted through 5 steps including literature review, expert focus group, Likert scale survey, three rounded Delphi study, and content validity ratio testing.

**Participants**

Overall, 36 participants were invited and contributed to the study. Two of them participated only in the NGT, thirteen were present in NGT, Delphi and Likert survey and twenty one participated in Delphi and Likert study stages. The fifteen experts present in NGT included 10 Ph.D candidates (all of the Ph.D students in southern Iran) and 5 faculty members (all of the active faculty members of medical ethics department), the majority (66%) being between 31 to 50 years of age. Some of the experts had a history of management at different levels of the health care system including the presidency of the University of Medical Sciences, vice chancellery for research, vice chancellery for education, vice chancellery for treatment, vice chancellery for health, the dean of city health network, the dean of the emergency care in several provinces of the country and the manager of the hospital. One of them had a history of working in the national TV as a journalist and executive/presenter for developing people’s information about health system. They had a history of working in fifteen cities of six provinces in the country; some with a history of working in only one city and some in different cities. As mentioned, totally 36 experts participated in the Delphi and the Likert scale survey:  8 Ph.D candidates and 12 faculty members (from medical and nursing schools), 10 ethics educated nurses and 6 other medical and paramedical professionals who had at least a course of ethics education and had paper and research history in the field of ethics.

Thirteen of the Delphi and Likert scale survey experts participated in the NGT, too. The Delphi experts were from different medical, nursing and paramedical fields such as pathology, paediatrics, ophthalmology, psychiatry, General Surgery, internal medicine, neurology, community medicine, legal medicine, medical education, nursing education, and nursing management, all affiliated to Shiraz University of Medical Sciences. Some of the experts had a history of management at different levels of the health care system including the presidency of the University of Medical Sciences, vice chancellery for research, vice chancellery for education, vice chancellery for treatment, vice chancellery for health, the dean of city health network, the dean of the emergency care in several provinces, the manager of the hospital, senior nursing officer, nursing supervisor, and dean of the nursing office. Also they had a history of working in 21 cities and 7 provinces in the country. The details of the Delphi participants’ demographics are shown in Table 1.

**Table 1:** Demographic information of the Delphi participants

**Variables**	**Frequency**	**Percentage**
Age-group in years (N = 36)		
20-30	4	11
31-40	10	28
41-50	17	47
51-60	4	11
> 60	1	3
Sex (N = 36)		
Female	17	47
Male	19	53
Position as professional practitioners (N=36)		
Physician	20	55
Pharmacist	2	5
Nurse	10	28
Operation room technician	1	3
Diagnostic laboratory technician	1	3
Medical jurisdiction expert	1	3
Medical management expert	1	3
Years of professional practice (totally 617 years, 34 experts)		
≤ 10	6	18
11-20	11	32
21-30	15	44
> 30	2	6
Years of practice as health care manager (totally 254 years, 23 experts)		
≤ 10	8	35
11-20	9	39
21-30	6	26
Years of practice as medical ethics activist and/or lecturer (totally 81 years, 15 experts)		
≤5	5	33.3
6 – 10	5	33.3
11-20	3	20
21-30	2	13.3
Academic Degree (N=12)		
Instructor	1	8
Assistant professor	3	25
Associated professor	5	42
Full professor	3	25
Participation in hospital ethical committees (N = 36)		
Yes	28	78
No	8	22

Informed consent forms were prepared and obtained from the participants in all stages of the study separately.  

**1. Literature review**

The researchers conducted an explorative literature review to find possible similar studies and the major domains for evaluating hospital ethics and health care organizational ethics. This review helped authors to make a better design for the research and also describe the different important domains in the matter from different perspectives of different experts all over the world. SAE, MA, and MS explored and reviewed the literature on the topic through search engine websites and scientific databases such as Scholar Google, Scopus and Pub Med by searching different combinations of some key words such as ethics, hospital, health care, organizational ethics and accreditation, and joined together for 7 meetings and discussed the issue to share the information aiming to propose the plan of the research after consulting SZT, step by step. Additionally, MA, and MS reviewed Pub Med and Scholar Google with the NGT and Delphi method in title and abstract and held 3 meetings by AM, and ZK to design the NGT and Delphi stages of the study.

**2. Expert Focus Group (Nominal Group Technique)**

The approach used in this step of the study was the use of Nominal Group Technique (NGT) along with NGT since it is more structured than the focus group design. NGT was established by Andrew H. Vande Ven and Andre L. Delbecq in 1968. It is defined as a group decision making method for creating ideas and identifying problems. In this technique a prioritized list of concepts are developed through multi voting and brainstorming by team members. The leader is accountable for guiding the brainstorming sections and recording the ultimate list of concepts based on the ideas of the group members (30). NGT is a good method to obtain group consensus, particularly when various individuals are involved in designing a model and the list of output is too long and thus has to be prioritized (31-33).

**Conducting the NGT activity**

The NGT meeting was held in 2012 October, and was attended by 15 medical ethics experts in southern Iran including 10 Ph.D students who were physicians or pharmacists (MD or PharmD) and 5 faculty members of the medical school with teaching experience of medicine from 13 to 30 years and that of medical ethics from 13 to 20 years, their academic degrees ranging from instructor to full professor. Authors excluded the medical faculty members who were former medical ethics teachers, and did not have any ethical lecture for more than 1 year. All of the 15 experts were invited to the Medical Ethics department conference hall at Shiraz University of Medical Sciences (School of Medicine). At first, authors used a self-administered questionnaire. The questionnaire asked for participants’ demographic information including age, gender, position, the participants’ last academic degree, years of medical practice, years of practice as medical ethics activist (lecturer, post graduate student, ethical committee member), and years of other special activities in health care system, as a professional, researcher, teacher or manager. Authors ensured the participants about the confidentiality of the information they provided. Index cards were prepared for all participants. The leader explained member roles and group aims by a kind welcome, a statement of the significance of the task and importance of each member's involvement and a suggestion that how the groups' output will be used.

NGT was performed through the following steps:

A question was written on the board as an issue statement *"what are the most important domains for ethical evaluation of a hospital?"* so that all members could see it. After that we had silently brainstorming ideas; members were allowed 5-10 minutes to write all their responses. So each idea was thoroughly discussed, members being encouraged to share ideas. Each person stated one idea at a time and his/ her idea was written on the board without any value judgment. For elaborating on the ideas on the board, each idea was discussed. Members, both pros and cons, were encouraged to share thoughts about each item. There was further clarification of each item so that everybody in the group might appreciate it well. In these stages, duplications were deleted. However, mixing two or more ideas into one was not done in the above mentioned stages. The participants were invited to rank order the top ten alternatives based on importance, clarity, and measurability: five as the most significant and one as the least significant. This was done by having members write the idea in the center of a card and their ranking on the right corner of the card.  The leader collected the cards and mentioned each ranking. The ranks for each item were averaged. All domains with their ranks were written on the board so that all members could see them. The ranked domains were further elaborated by the leader to guarantee that all participants had grasped what each item meant. Regarding the clarity and quantifiability of each domain, each member was again asked to rank the domains based on Likert-scale from five to one (five as the highest and one as the lowest). This was done silently and independently as in early ranking step. This ranking was limited to ten items per person. The rankings were again averaged. For the final ranking discussion, the group reviewed the ranking and discussed the outcome of the activity. In the end, all ranked priorities were listed in a table, beginning with the domain which ranked the highest. A column included the total votes for each item with the number of persons who voted for that item in front of the priority. Having gone through this, the 15 experts determined the list of priorities. Voting was based on five point Likert-scale. The meeting continued about 8 hours and it took about a week to finalize the experts’ ideas via interviews.

According to the experts’ scores, there were 3 scores for each domain which were from 1 to 5. After computing the mean of the points, the authors curved the points to the percentage. Then 15 top domains were distinguished. 

An advanced literature review discussion performed by SAE, MA and MS, and the domains found to be a part of the others were omitted. Finally, 11 main domains remained. Relevant indicators for these domains were listed in 11 tables with considering their face validity. The matter of the Delphi study was these 11 tables. 

**3. and 4. Delphi and Likert scale survey**

After identifying the domains, the research team applied another qualitative method to develop some objective indicators from these general and vague domains. In spite of some common members, the expert group members were not identical to the NGT experts. All NGT experts were invited to the Delphi. In addition, some other clinical professionals, especially doctors and nurses who had passed at least one course about nursing or medical ethics and had a history of hospital management experience or had publications in the field, were added to the experts. As Sinead Keeney et al. argue, individuals with only some knowledge in a specific field cannot be considered experts. Furthermore, the experts’ commitment to participating makes them more involved in the matter (34). For the Delphi survey a questionnaire was designed including 11 proposed domains and some of the objective indicators for each domain were designed preliminary by the authors. Also a 5 point Likert scale questionnaire was attached to each table indicator. In the first Delphi round, the questionnaires were sent to the experts.  They were asked to rank the list of domains and indicators from 1 (the lowest priority) to 5 (the highest priority).In the second Delphi round the questionnaires were analyzed and the mean and standard deviation scores were calculated. The respondents whose scores were significantly different from the mean score of the total group were asked to review their responses and re-evaluate their answers. The respondents could keep their initial scoring or change them, but they were asked to clarify their decision in this regard. The respondents were allowed to mention other domains and indicators they thought to be important but not listed. In the third Delphi round the last version of domains and indicators were sent to the experts for possible minor changes. The data were analyzed, using SPSS 14. Then, we prepared the final list of domains and indicators according to these experts’ opinions.

**5. Content validity measurement**

Finally, the authors performed content validity measurements according to Zaman Zadeh et al.’s recommended method. As Zaman Zadeh et al. maintain, the content validity of a study tool could be shown by using panel experts consisting of content experts and lay experts. The authors invited 25 clinicians including physicians and nurses who were not affiliated to the medical ethics department or hospital ethics committees and had no ward or hospital management history, but research experience or work in their professional fields as lay experts. In fact they were potential subjects for the product of this research. Also all of the Delphi study experts were invited to participate in content validity computing as content experts because they had experiences and work in health care ethics field. Two questionnaires were emailed to the experts; the first the ID questionnaire and the second the 71 indicator instrument produced in the study. They could score every indicator for its necessity in a three-degree range of “not necessary, useful but not essential, essential”. The scores varied between -1 and 1. Content Validity Ratio (CVR) was computed through Lawshe formula which is: CVR = (N_e_ – N/2)/(N/2), in which the N_e_ shows the number of panelists pointed “essential” and N shows the total number of panelists. The CVR value was computed for each indicator. A minimum CVR of 0.49 was required to retain an indicator in the final form of the instrument (35).

The ethics committee of the Vice chancellery for research at Shiraz University of Medical sciences approved the proposal. 

## Results

After discarding repeated domains during the NGT meeting, authors had 65 domains. In the second ranking step we had 43 domains and in the final ranking discussion they decreased to 34 domains.

The major domains driven are listed in Table 2 after performing NGT according to the total score which is the consequence of a combination of the experts’ points of view about the importance, clarity and measurability of the domains.

**Table 2:** The major domains determined by NGT

**No.**	**The domain**	**Total Score** **(/100)**	**The total mean point** **(/5)**	**The mean point for importance** **(/5)**	**The mean point for clarity** **(/5)**	**The mean point for measurability** **(/5)**
**1**	Informed consent	90.00	4.5	4.71	4.36	4.43
**2**	Medical confidentiality	89.40	4.47	4.71	4.57	4.14
**3**	Physician-patient economic relations	87.00	4.35	4.64	4.21	4.21
**4**	Respect for the patient’s autonomy	86.60	4.33	4.93	4.21	3.86
**5**	Ethical consultation policy in the hospital	86.20	4.31	4.57	4	4.36
**6**	The protocol of breaking bad news	83.80	4.19	4.29	4.21	4.07
**7**	Hospital ethics charter	83.40	4.17	4.5	3.86	4.14
**8**	Regulatory and control system	82.80	4.14	4.36	4.14	3.93
**9**	Respect for the patient’s rights	82.40	4.12	4.57	3.57	4.21
**10**	The existence of guidelines for usual moral dilemmas and ethical conflicts	80.60	4.03	4.29	3.79	4
**11**	Clinical ethics committee	80.40	4.02	4.43	3.71	3.93
**12**	Hospital approach to medical errors and malpractice	78.60	3.93	4.5	3.86	3.93
**13**	The spiritual care unit programs in the hospitals	78.60	3.93	4.14	3.71	3.93
**14**	Communication skills between personnel and professionals	76.20	3.81	4.57	3.64	3.21
**15**	Equitable accessibility to the basic medical care	74.80	3.74	4.14	3.57	3.5
**16**	Relevant continuing education for the personnel	73.40	3.67	3.64	3.43	3.93
**17**	Sufficient and appropriate patients’ accessibility to the information they required	72.40	3.62	4.14	3.57	3.14
**18**	The existence of positive role model professionals in the hospital	71.40	3.57	4.21	3.29	3.21
**19**	The patients’ social and cultural preferences	71.00	3.55	4.29	3.29	3.07
**20**	Concerns about ethics in the medical education (in educational hospitals)	70.80	3.54	4.21	3.21	3.21
**21**	Clarity and predictability of the structures and processes	70.40	3.52	4.14	3.21	3.21
**22**	Training and teaching hospital personnel about professionalism	69.60	3.48	4.36	3.14	2.93
**23**	Respecting patients’ visitors	69.00	3.45	3.93	3.14	3.29
**24**	Moral sensitivity in the managers’ viewpoints	68.00	3.40	4.21	3.29	2.71
**25**	The rate of medical lawsuits against wards, personnel, managers and physicians	67.20	3.36	3.36	3.14	3.57
**26**	Honest and trustful communications	66.20	3.31	4	3	2.93
**27**	Disaster management guideline	65.60	3.28	3.71	2.71	3.43
**28**	Emotional control skills (personnel psychiatry support)	63.80	3.19	3.79	2.71	3.07
**29**	Organizational happiness and friendship between personnel	62.40	3.12	3.79	2.64	2.93
**30**	Rights, interests, responsibilities and duties for all stake holders	62.00	3.10	3.93	2.5	2.86
**31**	Personnel’s team work	61.40	3.07	3.71	2.43	3.07
**32**	Personnel’s job satisfaction	60.00	3.00	3.71	2.57	2.71
**33**	Considering ethics as a major strategy in the hospital	58.60	2.93	3.93	2.5	2.36
**34**	Good intersectional relations	54.80	2.74	3.64	2.07	2.5

After the 3-phase Delphi was carried out, there were 11 tables of the indicators which appear in table 3. Meanwhile, an instruction on how to evaluate and complete the tables, approved by %70 of the experts, was prepared for each table. In this instruction, the whole points for each indicator and the method of scoring each indicator are explained. Some more details of the instruction are in the parentheses in front of the indicators in table 3.

As seen in the table 4, the 71 objective indicators, i.e. the study goal, were derived from 11 domains of Informed Consent, Medical Confidentiality, Doctor-Patient Economic Relations, Clinical Ethical Consultation Process in the Hospital, Patient right charter, Communication skills, Breaking Bad Medical News, Equitable Accessibility to Basic Medical Care, Hospital ethics committee, Hospital Ethical charter, and Hospital Spiritual and Palliative Care.

**Table 3:** The 11 major domains and 71 objective indicators determined finally

**Domain**	**Indicator **
**Informed consent**	1. Does the most knowledgeable member of the treatment team provide information to the patient or the proper surrogate decision maker?
	2. Is the information given to the most relevant person?
	3. Is the person who obtains the informed consent for the patient the most appropriate person?
	4. Does the patient give the consent voluntarily?
	5. Are the relevant ward personnel aware of the right form of taking informed consent?
	6. Are there any forms to know the decision of the patients who might get into conditions which make them unable to decide about their care?
	7. Does the patient have access to handouts, pamphlets, brochures, compact CDs or any other types of guidelines about the most prevalent disease in the ward?
**Medical confidentiality**	1. Has the patient’s diagnosis or any patient’s secrete information been recorded somewhere not visible to the public?
	2. What is the process of confidentiality about specific diagnoses such as HIV, and Hepatitis B and C?
	3. Do the clinicians, nurses and the related personnel consider confidentiality when obtaining informed consent?
	4. Do the clinicians, nurses and the related personnel consider confidentiality when breaking bad news or medical errors?
	5. Do the clinicians, nurses and the related personnel know who can legally have access to the patient’s records?
	6. Are the ward structure and the arrangement of beds and the location of patients appropriate to follow confidentiality?
	7. How much are the related ward professionals aware of confidentiality?
**Doctor-patient economic relations**	1. Are different types of the therapeutic professionals’ conflicts of interest determined in every ward by the ethical committee?
	2. Is there a regular monitoring plan for managing conflicts of interest between the therapeutic professionals and the patients in every ward approved by the hospital ethics committee?
	3. Is there any policy to watch and control illegal request for money or other interests by the treatment team?
	4. Parallel to the above watching efforts, is medical tariff reasonable?
	5. Are there any recorded cases of legal punishment for those who got or requested money (or other interests) illegally?
	6. In case of fee splitting, is there any approved process to control or manage it?
	7. In case of inevitable conflicts of interest between the treatment team and the patients, are the patients informed honestly and clearly?
	8. Regarding the insurance structure of the patients (Capitation, Fee-for-service …), are the ethical considerations of this type of insurance set and managed properly?
**Clinical ethical consultation process in the hospital**	1. Are there any recorded documents about the presence of the right advisors on the ward for ethical consultation round the clock?
	2. Are there any recorded documents of ethical consultation about hospital macro management such as budgeting and resource allocation?
	3. Are there any recorded documents of ethical consultation when two wards have conflicts on the admission of a patient?
	4. Are there any recorded documents to show the ethical consultation in case of conflicts of interest, ethical dilemma, taking informed consent, breaking bad news, confidentiality, surrogate decision maker, and allocation of resources (in intensive care units, transplantation, medication, etc.)?
	5. Are there any guidelines approved by the hospital ethical committee for the most common ethical consultation indications?
**Patient right charter**	1. Are the minimum appropriate, affordable services developed and approved in the ward?
	2. Are such services monitored by the head of the ward?
	3. Are patients informed effectively and sufficiently about their disease?
	4. Are patients informed effectively and sufficiently about the services process they receive?
	5. Is the patients’ right to choose their own doctors or treatment team respected?
	6. Are there any recorded documents that all patients in the hospital have access to a comprehensive complaint system?
	7. Can patients voluntarily share in the process of their disease diagnosis and treatment decision making?
	8. Is the patients’ privacy (physical, decision making, intimate, and proportional, informational) respected on this ward?
**Communication skills**	1. Are there any recorded documents about constant and regular education of communications skills to the personnel involved in patients and their companions’ issues?
	2. Are there role models, both scientifically and behaviorally, for effective communication in the ward?
	3. Is there the required psychological/psychiatric support to educate and train the personnel involved in patients and their companions’ issues on different emotion management skills?
	4. Are there any recorded documents of regular (annual) monitoring of attempts to promote the personnel behavior regarding maintenance of communication skills and ethical attitudes such as honesty toward patients, trustfulness, compassion, empathy and respect in the ward?
	5. Are there any recoded documents of regular (annual) monitoring of attempts to promote behavior based on respect, happiness and intimacy among the personnel of different ranks?
	6. Are there any recorded documents of regular (annual) monitoring of the attempts to promote interdepartmental relationships?
	7. Are there any recorded documents of regular (annual) monitoring of the attempts to promote the personnel job satisfaction in the ward?
**Breaking bad medical news**	1. Is there a proper location (room) in the ward to break bad medical news to the patients or their relatives?
	2. Does the most proper member of the treatment team, the most knowledgeable or the other trained staff approved by the ethical committee, break the bad medical news to the patients or their surrogates?
	3. Is there a guideline approved by the ethics committee for the personnel?
	4. Are all related personnel aware of the ward protocol about breaking bad medical news?
	5. Do the related personnel have access to the educational aids (written, audio-visual, and scientific multimedia) for the education of ward protocol?
**Equitable accessibility to basic medical care **	1. Are the basic medical care services on the ward, which must be accessible to all, clearly defined and approved?
	2. Are there any recorded documents about regular monitoring of patients’ access to those basic medical cares?
	3. Are the internal and external affordable capacities in the health system and hospital properly used to provide more equity for the catastrophic health care expenditure (especially for the needy patients)?
	4. Are there any recorded documents of a clear and regular plan to discover and prevent racial, ethnical, and national discrimination?
	5. Are there any recorded documents of a clear and regular plan to discover and prevent sex discrimination?
	6. Are there any recorded documents of a clear and regular plan to discover and prevent religious, political, or other types of discrimination, e.g. the level of education, or patients’ income?
	7. Has a clear process been approved and implemented for just allocation of different type of resources for all needy patients?
	8. Are there any recorded documents of a clear and regular plan to discover and prevent the probable misuse by powerful people (scientific, political, religious, economic, military, or professional) in order to achieve the more qualitative medical care?
**Hospital ethics committee**	1. Are the required guidelines for common moral dilemmas developed and announced?
	2. Are the approved and implemented guidelines comprehensive, diverse and general enough to fulfill the required guidelines for the other tables?
	3. Have the ethics committee members received the required training on their responsibilities?
	4. Are the implementation and effectiveness of the approved guidelines evaluated and monitored through evaluation of patients’ attitudes toward their satisfaction of the delivered services?
	5. Is the implementation of international and national ethical codes in medical education, treatment and research in hospitals monitored and the violation cases traced and managed properly?
**Hospital ethical charter **	1. Has an updated and comprehensive ethical charter been approved by the hospital board of managers?
	2. Has the hospital ethical charter priority to the hospital annual budget?
	3. Are the different issues of medical ethics including the items of other hospital ethical accreditation standards considered in this charter?
	4. Are clinical ethics committee, medical research ethics committee and other responsible committees for mortality and morbidity conferences and medical errors considered in the hospital ethical charter?
	5. Is the issue of ethical equitability in health care delivery considered in the compilation of the hospital ethical charter?
	6. Is there an ethical audit process for intra-organizational and mandated laws in the hospital to detect the conflicts and recommend suggestions for improvement?
**Hospital spiritual and palliative care**	1. Has the hospital spiritual care unit set up and approved a process for the patients’ spiritual needs analysis and implemented spiritual cares by trained professional people?
	2. Are there any palliative and end stage cares for patients with terminal diseases?
	3. Are there facilities such as hospice in the hospital or affiliated with the hospital to take care of the patients without any indications to be hospitalized on the wards or kept at home?
	4. Is the hospital chaplaincy unit active to support needy patients?
	5. Have the chaplaincy and spiritual care units set up and implemented an active process to deliver proper services to vulnerable people?

After conducting NGT and Delphi, as mentioned CVR was computed for each indicator, the CVR scores shows in table 4.

**Table 4:** Content validity ratio scores computed for each indicator

**No.**	**Domain/ indicator number**	1	2	3	4	5	6	7	8
**1**	Informed consent	0.90	0.95	0.64	0.95	0.90	0.36	0.64	-
**2**	Medical confidentiality	0.95	0.90	0.95	0.95	0.90	0.90	0.82	-
**3**	Ethical charter	0.9	0.55	0.82	0.73	0.82	0.95	-	-
**4**	Patient right	0.64	0.73	0.73	0.95	0.73	0.95	0.95	0.82
**5**	Economic relations	0.82	0.73	0.73	0.73	0.64	0.36	0.82	0.73
**6**	Justice	0.64	0.55	0.55	0.55	0.73	0.73	0.73	0.73
**7**	Communication skills	0.55	0.73	0.90	0.64	0.82	0.55	0.82	-
**8**	Bad news	0.64	0.82	0.95	0.82	0.55	-	-	-
**9**	Spiritual care	0.73	0.9	0.64	0.95	0.82	-	-	-
**10**	Ethical consult	0.73	0.55	0.55	0.82	0.82	-	-	-
**11**	Ethics committee	0.90	0.64	0.82	0.82	0.73	-	-	-

## Discussion

The research goal was to develop an instrument to accredit hospital ethics. After performing four steps, 71 indicators of 11 domains were achieved. These domains included Informed consent, Medical confidentiality, Physician-patient economic relations, Ethics consultation policy in the hospital, Ethical charter of hospital, Breaking bad medical news protocol, Respect for the patient’s rights, Clinical ethics committee, Spiritual and palliative care unit programs in the hospitals, Healthcare professionals’ communication skills, and Equitable access to the healthcare. The majority of the domains and indicators are present in some other valid organizational ethics in health care (13, 14); however, they have been set according to ethical problems in the Iranian hospitals and compatible to the Iranian culture. So they are originally in Persian language and some indicators are set differently.

The hospital management issues on the medical ethics agenda are not valued as they deserve. However, management decisions taken in the field of hospital care are actually riddled with ethical questions and do have an important impact on patients, staff, and the community being served. As a result, ethical aspects of hospital management require more attention and inspection than they have received up to now (36). The question arising here is what should be considered in this regard. Results showed that “Respect for patient autonomy” is the most important domain to evaluate hospital ethics followed by the way in which “Informed consent” is obtained and “Medical confidentiality” concerns. Other important domains were “Doctor patient economic relations”, “Clinical ethical consultation process in the hospital”, “Patient right charter”, “Professional personnel communication skills”, “Breaking bad medical news strategy in the clinical wards”, “Hospital spiritual and palliative care methods and processes”, and “Equitable accessibility to basic medical care”. All these domains must be monitored via “Hospital ethics committee” plans and managements, as some of Delphi experts mentioned and the others adopted. Those plans could exhibit in “Hospital ethical declaration”. Ethics committee is the responsible structure of the hospital to monitor the execution of the plan. These domains are, in fact, the most important domains to accredit hospitals ethically.

Authors were looking for some indicators for general hospital ethics program promotion based on Iranian cultural considerations. So indicators are the tools to accredit purely ethical processes as an organizational behaviour in a general clinical climate (e.g. the behaviour of surgery ward about the confidentiality process).

The authors think organizational standards could focus on patient rights or patient safety or improvement of the quality of care doctors or nurses provide, even if these standards don’t meet organizational behaviour in the specific field of medical ethics. In other words, a hospital is an organization similar to other organizations and has clients known as “patients”, so hospitals have ethical responsibility to their clients. It is, in fact, the general ethical responsibility of all organizations against their clients. We tried to focus on purely ethical standards to improve hospital bioethical behaviour in addition to the general organizational behaviour. So the authors think that there are some additional bioethical indicators to transform accreditation into ethical accreditation.

Potter (6) holds that organizational ethics focuses on marketing and money, something strange for those who think ethical matters in hospitals are Informed Consent and Advanced Directive, and so on. The study indicators have the capacity to integrate organizational ethics and hospital ethics. The economic relationship between doctors and patients, as well as the Informed Consent process as an organizational behaviour of the hospital wards are important. In the NGT study respect for autonomy became the most important domain; however, the total score of Informed consent was the first. Autonomy is also an important factor introduced by World Health Organization in response to inpatient and outpatient services (37).

Beauchamp and Childress believe the concept of autonomy belongs to individuals’ decision making in health care and research as patients and research cases (2); however, some of the experts think the domain of autonomy is not limited to the patient. They maintain that autonomy applies to both patients and doctors (38). Experts’ emphasis was on the patients’ autonomy and it was the most important domain for improving hospital ethics. Rashidian et al. also showed that although autonomy was rated by Iranians as an important part of responsiveness in hospitals, it was scored as the worst performing domain based on people’s experiences in Tehran hospitals (39). Beauchamp and Childress also maintain that the principle of respect for autonomy has both negative and positive aspects and some of the ethical codes arise from the principles such as: telling the truth, respecting privacy of others, protecting confidentiality, obtaining consent for interventions, helping others, and making important decisions if required (2). It seems that different indicators have not the same priority; some are the base and root and the others the consequence and the fruit. Further inquiries seem to be essential to reach exact and objective indicators. Confidentiality is one of the most dilemmatic ethical responsibilities of the medical professionals (40). It can influence all of the steps of establishing good medical practice such as making trustful therapeutic relations, history taking, and improving patients’ compliance for following the prescription orders (41).

Although experts made a distinction between Informed consent and the principle of autonomy, some think Informed consent is the doctrine of the principle of autonomy and has a central role in the physician-patient relationship (38, 42). Vick and Scott think doctor-patient economic relationship has significant effects on patients’ decision making and satisfaction (43). The question arising here is that what should be considered in this connection. Results showed that Respect for patient autonomy is the most important domain to evaluate hospital ethics. The way in which Informed consent is obtained and Medical confidentiality concerns are both in the second place. Other important domains are Doctor patient economic relations, Clinical ethical consultation process in the hospital, Patient right bill, Professional personnel communication skills, Breaking bad medical news strategy in the clinical wards, Hospital spiritual and palliative care methods and processes, and Equitable accessibility to basic medical care. All of these domains must be conducted via Hospital ethics committee plans and managements. These plans could be manifested in Hospital ethical declaration. Ethics committee is the responsible structure of the hospital to monitor the execution of the plan. Those domains are the most important domains to accredit hospitals ethically.

Economic relationship between medical care providers and patients is, in fact, at the top of the list. The clarity of the economic decisions, knowing conflict of interest, avoiding or disclosing that to the patients are some important issues in the biomedical ethics literature (44, 45). Also different patients’ insurance systems and the methods of referral therapeutic systems or health networks in the country could have valuable impacts on biomedical ethics in hospitals. When there is fee for service insurance system (46), the ethical considerations could be different from when the insurance payment system is something like capitation (47, 48). Puma and Darling believe that patient care could be improved by the ethics consultation system in the hospital (49). Yen-KoLin et al. argue that ethics consultation on a medical ward could increase patients’ satisfaction and cause major improvements in their privacy (50). Nowadays more than 81% of all hospitals in the United States (US) have some form of ethics consultation facility to report complex ethical issues (51). Business ethicists think the most important criterion for developing ethics in every organization is the existence and implementation of the ethical bill including organization code of ethics. Collins and Porras believe any effective ethical declaration for every organization including hospitals or other businesses must represent the core ideology of the institute, which, in turn, is made up of two distinct elements: core values, a system of conducting principles and doctrines, and core purpose, the most fundamental cause for institutional existence (52). Nowadays patients want to know about their disease even if the information would contain bad news, so we could say breaking rather than withholding bad news could be presumed as a criterion for ethical development in a hospital (53). The next indicator is hospital regulatory system, which is the first chapter of Wu et al.'s criterion for hospital ethical accreditation. Wu et al. apply that system to regulate and manage therapeutic fields (14), while Yan and Munirrelate regulatory system is applicable to the medical research (54). The question authors must answer in further inquiries is “What are the differences and similarities of this domain and the domain of hospital approach to medical errors and malpractice?” Perhaps it can be said that hospital approach to medical errors could be a criterion for good regulatory system in a hospital. Some medical ethicists think full disclosure is mandatory for the medical team if medical error or malpractice has occurred. Lawsuits decreased after employing such ethical codes (55). Hashemi and her colleagues suggest that reporting medical errors is an important way to improve patient safety in Iran (56). Hamasaki and Akihito Hagihara found that when the professional members of medical team, especially physicians, disclose a condition to the patients and do their explanatory duty before treatment or surgery, it could decrease the rate of legal problems (57). Delbanco et al. report that in 1996 the US federal Health Insurance Portability and Accountability Act (HIPAA) authorized the patient rights society to appraise the archives and make the required amendments. Shortly afterward, the Institute of Medicine made the society assess the note not as a static object, but as a living, interactive document communal among patients and providers (58). The distinguishing border between the patient autonomy and the patient rights may be confusing. Perhaps, it can be said that respecting the patient’s autonomy is considered one of the most significant patients’ rights. Last year Iranian Ministry of Health and Medical Education codified and implemented the patient rights charter (29). Ethical committees could solve conflicts that may arise between patients and medical care providers, especially when the conflict is based on the differences between their values and expectations (10, 59). As Potter has pointed out redefining hospital ethics committee roles will help health institutions to take more responsibility in the integrated ethics program which is the second stage of the bioethics evolution. This work shows that Iranian medical ethics experts who participated in the study, as well as Potter, consider a new role for hospital ethical committees, i.e. they should conduct hospital ethical internal and external evaluation. However, there are not enough ethicists to serve as consultants and core members of these committees all over the country; it is hoped to achieve this level soon (6). Providing medical care involves different levels of management from individual patient’s situation management level to the world health management level. Every level of management might face significant numbers of moral dilemmas. Hospital managers, ethical consultants or committees, for example, could apply a tool for guiding professionals to manage moral dilemmas. It could not only solve ethical problems, but also decrease moral distress in medical professionals (60). The existence of such guidelines or tools for usual moral dilemmas and ethical conflicts suggests the importance of the ethical issues to hospital mangers and it may be considered an achievement of the ethicists’ efforts in the system. Parallel to their medical needs, patients need spiritual care. Providing spiritual and palliative care can meet such a need which is a psychosocial demand for patients throughout their life (61-63). Gross believes communication skills are important competencies needed in order to be a good ethics instructor and a successful medical professional. He asserts that it is relatively uncontroversial that communication has an important impact on physicians’ and other professionals’ behavioural outcome (64). Zarei and colleagues investigated the Iranian patients' expectations from the hospital personnel, nurses and practitioners. The purpose was to improve their empathy dimension in both public and private hospitals. They recommend that medical professionals make their patients aware of their disease situations, answer their questions, be familiar with and pay attention to their emotional and social needs and be on hand when needed (65). Equitable accessibility to the basic medical care was the 15^th^domain. In fact, the matter of equity and the justice is one of the 4 principles of Beauchamp and Childress (2). Resource allocation in health care system is one of the most important ethical issues. At least, there are three levels for allocation. In the national level we can negotiate about allocating resources to healthcare rather than the other social needs in the country. Resource allocation within the healthcare sector could be the second level, and allocating resources among individual patients is the third. Distributive justice and avoiding different types of discrimination are of great importance (66). Gonzalez-Block‘s study showed that "Equity" was one of the most important domains deserving attention and must be considered in health care policy making in developing countries (67). One of the strengths of the study was to provide some crucial and good indicators for assessment and their application to the 40 district public hospitals in Fars Province in southern Iran to improve ethical levels in those hospitals. In the second step, 14 private and 5 public hospitals in Shiraz not affiliated to SUMS could be the target. Such ethical issues can, of course, be used in all hospitals all over the country. Among the most important strengths of the present project is the fact that these experts are the system owners. They are all working in the relevant fields and all have the opportunity and responsibility to consider the issue. It is obvious that the system owners could make better decisions for the system than outsiders who are not fully familiar with the system. Also they have the power for Implementation. Potter uses some concentric circles to show various levels of the bioethics including: Personal decisions, Family of support, Health team, Health agency, Delivery system, Societal context, International systems and biospheric systems. Indeed, this study tables are often in the field of Health Agency according to the classification. However, a few tables are also related to the Delivery system. There are also some indicators about the other levels, some of which in the field of clinical ethics prima facie. These indicators can be viewed from an organizational aspect. For example, we can ask if there is an approved protocol about obtaining Informed Consent in a clinical ward, that is a question related to the organizational aspect of an originally personal or health team matter. As potter recommended we “move back and forth to maximize interaction of the different levels (6). Suhonen et al. in their review article have shown that the majority of experimental studies on organizational ethics focused on acute hospitals or acute wards of the hospitals. However, these indicators consider the wards in a general hospital (7).

**Methodological considerations**

NGT is a structured qualitative research; this method plus three round Delphi could reveal the experts’ opinion in the best way, and it can be an excellent example or role model for the other medical universities and hospital managers in Iran and other countries.

Regarding the limitations and weaknesses, performing NGT and three round Delphi was a huge work and we couldn’t design any needs assessment before the beginning of the study. Maybe the study would have led to more significant findings if it was based on needs assessment rather than opinion survey. Although opinion survey is valuable, it has some limitations. For example, it is possible that we had some subjectivity in the results; however, the experts were the best participants we could have access to and we tried to do the best results by giving appropriate feedback to the experts. Other organizational ethics researchers in medical and nursing care services also use Delphi and NGT to explore ideas, and even producing instruments and evaluation tools (68-70).

The way in which the standards are applied and checked is also very important. For example, we can ask which organization is responsible for doing an internal evaluation, the hospital or health care managers or the Ministry of Health. Or it could be done as an external evaluation by NGOs or international organizations such as human right watching organizations to decrease conflict of interests between the providers of care and the ethical creditors. On the other hand, could we have a unique questionnaire in different countries with different cultures and different ethical norms? Can we say the main domains in the entire world are comparable but the standards arising from the domains can be somehow different according to the regional differences? What are the optimum questions to accredit ethics in the hospital? 

## Conclusion

The listed domains in the study seem to be the most important domains in evaluating hospital ethics and could be generalized to other medical settings. However, they could be enriched through further studies, considering needs analysis and cultural issues. Accrediting and/or ranking hospitals by such criterion could appreciate hospitals which are more ethical than the other, inform the patients about them, and make a move to improve medical ethics in the hospitals. National and international accreditation/ranking organizations can prepare annual ethical hospital ranking list and it could serve as a help devise for the patients to select their hospitals for common treatments or medical tourism. 
